# Effect of lung isolation with different airway devices on postoperative pneumonia in patients undergoing video-assisted thoracoscopic surgery: a propensity score-matched study

**DOI:** 10.1186/s12890-024-02956-4

**Published:** 2024-04-04

**Authors:** Hongyi Xiao, Huan Zhang, Jiying Pan, Fangli Yue, Shuwen Zhang, Fanceng Ji

**Affiliations:** Department of Anesthesiology, Weifang People’s Hospital, Kuiwen District, No. 151 Guangwen Street, Weifang, 261041 China

**Keywords:** Airway devices, Video-assisted thoracoscopic surgery, Postoperative pneumonia

## Abstract

**Background:**

Postoperative pneumonia is one of the common complications after video-assisted thoracoscopic surgery. There is no related study on the effect of lung isolation with different airway devices on postoperative pneumonia. Therefore, in this study, the propensity score matching method was used to retrospectively explore the effects of different lung isolation methods on postoperative pneumonia in patients undergoing video-assisted thoracoscopic surgery.

**Methods:**

This is A single-center, retrospective, propensity score-matched study. The information of patients who underwent VATS in Weifang People 's Hospital from January 2020 to January 2021 was retrospectively included. The patients were divided into three groups according to the airway device used in thoracoscopic surgery: laryngeal mask combined with bronchial blocker group (LM + BB group), tracheal tube combined with bronchial blocker group (TT + BB group) and double-lumen endobronchial tube group (DLT group). The main outcome was the incidence of pneumonia within 7 days after surgery; the secondary outcome were hospitalization time and hospitalization expenses. Patients in the three groups were matched using propensity score matching (PSM) analysis.

**Results:**

After propensity score matching analysis, there was no significant difference in the incidence of postoperative pneumonia and hospitalization time among the three groups (*P* > 0.05), but there was significant difference in hospitalization expenses among the three groups (*P* < 0.05).

**Conclusions:**

There was no significant difference in the effect of different intubation lung isolation methods on postoperative pneumonia in patients undergoing thoracoscopic surgery.

## Introduction

Video-assisted thoracoscopic surgery (VATS) is an important method for the treatment of pulmonary diseases. Compared with traditional thoracotomy, VATS has the characteristics of less trauma, faster recovery and shorter hospital stay [1, 2]. Although VATS has certain advantages, postoperative pulmonary infection still occurs, which affects the rehabilitation and medical quality of patients [3]. Postoperative pneumonia (POP) is the most common type of pulmonary complications after VATS. The incidence of POP in thoracic surgery is between 6.2% and 31.7% [4–7].

Lung isolation techniques are central to anaesthetic management for thoracic surgery. With thoracic surgery entering the minimally invasive era of accelerated rehabilitation, the indications and tools of lung isolation technology are constantly changing. The commonly used lung isolation techniques in clinical practice include double-lumen endobronchial intubation, tracheal tube combined with bronchial blocker, and the emerging lung isolation technique in recent years: laryngeal mask combined with bronchial blocker.

In VATS, laryngeal mask combined with bronchial blocker, as an emerging technology, is superior to double-lumen endobronchial tube in postoperative sore throat, hoarseness and airway injury [8, 9]. However, there is still a lack of relevant research on postoperative pulmonary complications. A current study has found that in non-cardiothoracic surgery, different airway devices have different effects on the impact on postoperative pulmonary complications in patients [10]. Compared with tracheal intubation, patients using laryngeal mask have fewer postoperative pulmonary complications. Therefore, this study retrospectively explored the effect of different intubation lung isolation methods on POP in patients undergoing VATS using propensity score matching (PSM) method.

## Materials and methods

### Setting and patients

The study was approved by the Ethics Committee of Weifang People's Hospital. The information of patients who underwent VATS in Weifang People 's Hospital from January 2020 to January 2021 was retrospectively included. Inclusion criteria included patients aged ≥ 18 years, with American Society of Anesthesiologists (ASA) classification I–III. Patients with severe cardiopulmonary disease; those with a change in surgical procedure; those transferred to the ICU postoperatively; those with preoperative pneumonia and those with some missing data were excluded. According to the airway management method during anesthesia, the subjects were divided into laryngeal mask combined with bronchial blocker group (LM + BB group), tracheal tube combined with bronchial blocker group (TT + BB group) and double-lumen endobronchial tube group (DLT group).

**Fig. 1:**
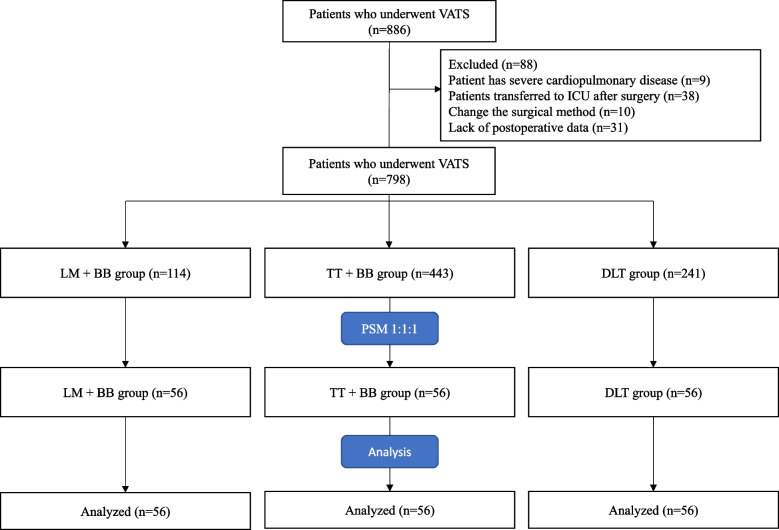
Flow chart showing the process used to select the study sample

From January 2020 to January 2021, a total of 886 patients underwent VATS in our hospital. According to the exclusion criteria, 798 patients were included in the study, including 114 in the LM + BB group, 443 in the TT + BB group, and 241 in the DLT group (Fig. 1).

### Anesthesia

Intravenous access and invasive arterial blood pressure were established after patients entered the operating room, and electrocardiogram, non-invasive blood pressure, pulse oximeter, PETCO_2_ and bispectral index (BIS) levels were monitored. The patient underwent lung isolation technique during anesthesia, and the following anesthesia protocol was used. Anesthesia induction was achieved with propofol target-control infusion (plasma concentration: 3.0 ~ 4.0 µg/mL), sufentanil (0.3 ~ 0.4 µg/kg), and rocuronium (0.6 ~ 0.9 mg/kg). After induction of anesthesia, the airway device was placed for airway management. After intubation is completed, the patient is placed in the healthy side position, and the axilla on the healthy side is appropriately elevated to increase the intercostal space on the operative side to facilitate surgery; the patient's upper limbs are suspended overhead to increase the surgeon's surgical space. Anaesthesia was maintained with propofol target-control infusion (plasma concentration: 2.2 ~ 2.5 µg/mL) and remifentanil (0.1 µg/kg/min), and rocuronium was added intermittently according to the needs of the operation. After starting the operation, an observation hole was made in the 7th intercostal space of the midaxillary line, about 1.5 cm, and an operating hole was made between the 4th or 5th intercostal space of the anterior axillary line, about 3.0 ~ 4.0 cm. The operator performed the operation in the incision hole, including the process of tissue dissociation, blunt dissection and hemostasis. An auxiliary hole was made between the 8th or 9th ribs of the posterior axillary line, about 1.0 cm. The resected specimens were placed in the bag and removed through the operating hole. According to the preoperative surgical planning and comprehensive evaluation of intraoperative conditions, systematic lymph node dissection or lymph node sampling was performed, and closed drainage tube was placed in the postoperative incision. Routinely close the chest and suture the skin.

In the LM + BB group, the laryngeal mask was inserted into the appropriate position, and then the bronchial blocker was inserted with the assistance of fiberoptic bronchoscopy. In the TT + BB group, a tracheal tube with an inner diameter of 7.0 mm or 7.5 mm was inserted under the guidance of a visual laryngoscope. Then, under the guidance of fiberoptic bronchoscopy, the bronchial blocker was placed into the corresponding bronchus. In the DLT group, the double-lumen endobronchial tube was inserted under the guidance of visual laryngoscope, and the position of the double-lumen endobronchial tube was examined by fiberoptic bronchoscopy.

### Data collection

Patient information such as age, sex, weight, preoperative albumin level, presence of underlying diseases (diabetes mellitus and hypertension), ASA classification, duration of surgery, intraoperative infusion volume, intubation method, new pneumonia diagnosis within 7 days after operation, hospitalization time, hospitalization costs and other patient information were obtained through the hospital information system. The main outcome was the incidence of pneumonia within 7 days after surgery; the secondary outcome were hospitalization time and hospitalization costs. Pneumonia is diagnosed with a suspected respiratory infection treated with antibiotics and at least one of the following: new or change of pulmonary opacities, white blood cell count > 12,000/mm^3^, body temperature > 38.5℃ and positive sputum culture [11].

### Statistical analyses

PSM was performed using a logistic regression model to mitigate the selection bias in the present study. The parameters used for PSM were age, sex, weight, preoperative albumin level, presence of underlying diseases (diabetes mellitus and hypertension), ASA classification, duration of surgery, intraoperative infusion volume. The three groups of patients were matched using a 1:1:1 nearest neighbor matching algorithm without replacement, with a caliper of 0.02 of standard deviation of the propensity score on the logit scale.

All measurement data were tested for normality and homogeneity of variance. Data that conformed to a normal distribution were expressed as mean ± standard deviation (x̄ ± S). One-way analysis of variance was used for comparison between groups. Data that did not conform to the normal distribution were expressed as interquartile range [M (Q1–Q3)], and rank sum test was used for comparison between groups. Count data were expressed as percentage, and chi-square (χ^2^) test was used for comparison between groups [12]. Statistical analysis was performed with the SPSS 25.0 software (IBM Corporation, Armonk, NY, USA) and R statistical software version 3.6.3 (R Foundation for Statistical Computing, Vienna, Austria). *P* < 0.05 was considered statistically significant.

## Results

### Comparison of the three groups of patients before propensity score matching

There were significant differences in age, ASA classification, duration of surgery, intraoperative infusion volume, incidence of postoperative pneumonia, hospitalization time and costs among the three groups (*P* < 0.05). However, there was no significant difference in gender, weight, preoperative albumin, extent of resection, and the prevalence of hypertension and diabetes (*P* > 0.05) (Table 1).

**Table 1:** Comparison of the data before PSM in the three groups of patients

	LM + BB group (*n* = 114)	TT + BB group (*n* = 443)	DLT group (*n* = 241)	*P-value*
Age (years)	54.61 ± 11.71	57.68 ± 9.90	58.15 ± 9.62	0.005
Male (%)	53.5	54.9	53.9	0.954
Female (%)	46.5	45.1	46.1	
Weight (kg)	62.80 ± 8.61	64.70 ± 10.20	63.97 ± 9.61	0.157
Preoperative albumin level(g/l)	43.89 ± 4.20	42.90 ± 4.62	43.12 ± 4.15	0.115
Diabetes mellitus(%)	10.5	12.0	11.6	0.913
Hypertension(%)	20.2	29.6	29.4	0.121
ASA classification(n)				0.032
II grade(n)	101	368	187	
III grade(n)	13	75	54	
Extent of resection(%)				0.788
wedge resection	7.0	9.9	10.8	
segmentectomy	7.9	8.6	9.5	
lobectomy	85.1	81.5	79.7	
Intraoperative infusion volume(ml)	1140.35 ± 381.30	1215.35 ± 386.89	1294.40 ± 370.33	0.001
Duration of surgery(min)	124.63 ± 46.17	140.18 ± 52.84	147.81 ± 51.33	0.001
Pulmonary infection(%)	14.0	24.4	25.7	0.038
Hospitalization time(day)	10(8–12)	10.5(8–14)	11(9–15)	0.018
Hospitalization cost(yuan)	49689.96(41181.49–57921.67)	52756.76(43916.71–60755.16)	54,797.52(45408.19–61,334.80)	0.010

LM + BB group: laryngeal mask combined with bronchial blocker group. TT + BB group: tracheal tube combined with bronchial blocker group. DLT group: double-lumen endobronchial tube group

### Comparison of the three groups of patients after propensity score matching

Due to the statistical differences in age, ASA classification, duration of surgery and intraoperative infusion volume among the three groups of patients, this study used the PSM method to match the three groups according to 1: 1: 1, and a total of 168 patients were successfully matched. The unbalanced covariates among the three groups reached equilibrium after PSM. There was no significant difference in the incidence of postoperative pneumonia and hospitalization time among the outcome indicators (*P* > 0.05), but there was significant difference in hospitalization costs among the three groups (*P* < 0.05) (Table 2).

**Table 2:** Comparison of the data after PSM in the three groups of patients

	LM + BB group (*n* = 56)	TT + BB group (*n* = 56)	DLT group (*n* = 56)	*P*-value
Age (years)	53.45 ± 11.16	55.25 ± 11.61	57.77 ± 10.40	0.119
Male (%)	55.4	58.9	57.1	0.930
Female (%)	44.6	41.1	42.9	
Weight (kg)	61.50 ± 8.04	64.20 ± 10.80	65.10 ± 8.81	0.107
Preoperative albumin level(g/l)	43.29 ± 4.38	43.44 ± 4.37	43.54 ± 4.50	0.957
Diabetes mellitus(%)	8.9	19.6	17.9	0.244
Hypertension(%)	17.9	25.0	25.0	0.580
ASA classification(n)				0.271
II grade(n)	52	51	47	
III grade(n)	4	5	9	
Extent of resection(%)				0.857
wedge resection	5.4	8.9	10.7	
segmentectomy	7.1	7.1	8.9	
lobectomy	87.5	83.9	80.4	
Intraoperative infusion volume(ml)	1130.36 ± 411.50	1212.50 ± 399.57	1276.79 ± 329.18	0.130
Duration of surgery(min)	123.95 ± 42.90	141.75 ± 49.32	134.09 ± 44.60	0.121
Pulmonary infection(%)	12.5	25.0	19.6	0.240
Hospitalization time(day)	10(8–12)	11(8–14)	11(9–13)	0.165
Hospitalization cost(yuan)	47761.58 ± 11653.38	55577.81 ± 14684.79	51130.83 ± 11548.10	0.006

LM + BB group: laryngeal mask combined with bronchial blocker group. TT + BB group: tracheal tube combined with bronchial blocker group. DLT group: double-lumen endobronchial tube group

## Discussion

In this study, the propensity score matching method was used to match the three groups of patients to obtain a balanced research cohort. In selecting the matching variables, general information about the patients (age, sex, weight), as well as preoperative albumin, ASA classification, duration of surgery, intraoperative fluid volume, and prevalence of hypertension and diabetes mellitus, which have a potential impact on postoperative pneumonia, were included in the equations in order to reduce the impact of these factors on the outcome. Finally, the results of the study showed that there was no significant statistical difference in the incidence of pneumonia within 7 days after surgery and the length of hospital stay among the three groups, but the group of the laryngeal mask combined with the bronchial occluder was lower than the other two groups in terms of the incidence of postoperative pneumonia, the length of hospital stay, and the cost of hospitalisation.

In thoracic surgery, double-lumen endobronchial tube is the most common method for lung isolation [13]. This traditional lung isolation technique provides good surgical conditions for the surgeon, but it also brings strong stress response to the patient, especially the increase of blood pressure and heart rate during intubation, as well as cough, bronchospasm, and postoperative pharyngeal discomfort after extubation [14, 15]. In order to avoid the related risks caused by the above double-lumen endobronchial tube intubation, some scholars began to use laryngeal mask combined with bronchial blocker to compensate for the shortcomings of double-lumen intubation [8, 9, 16]. Compared with tracheal intubation, the use of laryngeal mask can reduce the incidence of laryngeal-related complications and reduce the stimulation to the root of epiglottis. And the protective function of laryngeal mask for the throat may help to reduce postoperative pulmonary complications using laryngeal mask [17, 18]. In addition, laryngeal mask airway can also reduce the incidence of postoperative respiratory and circulatory system related complications, reduce the cough symptoms caused by extubation, greatly increase the comfort of patients, and is more conducive to the clinical recovery of patients after surgery.

In the field of thoracic surgery, there is no research on the correlation between laryngeal mask combined with bronchial blocker and postoperative pneumonia. However, in non-cardiothoracic surgery, the effect of laryngeal mask on reducing postoperative pulmonary complications has been confirmed compared with tracheal intubation. Yang et al*.* studied postoperative pulmonary complications in elderly patients undergoing elective noncardiothoracic surgery with tracheal intubation or laryngeal mask ventilation and found that patients in the laryngeal mask group had a lower incidence of postoperative pulmonary complications compared with patients in the tracheal intubation group [10]. In addition, a retrospective cohort study found that tracheal intubation was associated with an increased risk of emergency tracheal reintubation compared with laryngeal mask airway, and the incidence of postoperative pneumonia was higher in the tracheal intubation group [19]. Although the incidence of postoperative pneumonia in the LM + BB group in this study was lower than that in the other two groups, there was no significant statistical difference with the other two groups, which may be related to the disadvantage of bronchial blockers [20, 21]. The bronchial blocker is prone to intraoperative displacement leading to reexpansion of the affected lung, and there is also the problem of difficulty in suctioning the collapsed lung, which to some extent may increase the incidence of postoperative pneumonia [22].

Both double-lumen endobronchial tube and bronchial blocker have their own advantages and disadvantages, but which airway device is more beneficial to the prognosis of patients is still controversial. At present, a number of studies have confirmed that the use of bronchial blockers can reduce the incidence of airway injury and postoperative sore throat and hoarseness [23, 24]. In addition, a prospective randomized controlled study found that compared with patients using double-lumen endobronchial tube, patients using bronchial blocker had a lower incidence of pneumonia within one week after surgery and a better prognosis [25]. However, Lin et al. found that patients receiving bronchial blocker tended to have more severe lung infiltration (especially on the surgical side) and a higher incidence of ICU hospitalization [26]. In this study, although the incidence of postoperative pneumonia in the TT + BB group was the highest, there was no statistically significant difference between the three airway devices, which may be related to the main research direction, the basic condition of the patient and the proportion of different types of surgery.

As the concept of accelerated rehabilitation surgery spreads, nonintubated VATS is also coming to the fore. It has been reported that nonintubated VATS with spontaneous breathing can reduce patients ' throat discomfort and hospitalization time [27]. But at the same time, it can also produce a series of problems such as hypercapnia, acidosis, uncontrollable pain or body movement, and postoperative atelectasis [28]. Therefore, a less invasive and reliable lung isolation technique is more desirable. Perhaps with the increase of the application of laryngeal mask combined with bronchial blocker technology and the development of visual technology, the advantages of laryngeal mask combined with bronchial occluder for lung isolation will be gradually revealed, which is more in line with the concept of comfortable medical treatment.

Nevertheless, several limitations in our study are noted. Firstly, this study only retrospectively observed the incidence of pneumonia during hospitalization within 7 days after surgery, and did not collect the prognosis of patients after discharge. Secondly, because the laryngeal mask combined with bronchial blocker technology is one of the emerging anesthetic technologies, so the number of cases used is relatively small, and multicenter, large sample data are still needed to verify in the later stage.

## Data Availability

The datasets used in the present study are available from the first author and corresponding authors on reasonable request.

## Abbreviations

ASA: American Society of Anesthesiologists
PSM: Propensity score matching
POP: Postoperative pneumonia
VATS: Video-assisted thoracoscopic surgery
